# Chemical composition, energy and amino acid digestibility in double-low rapeseed meal fed to growing pigs

**DOI:** 10.1186/s40104-015-0033-0

**Published:** 2015-08-21

**Authors:** Peili Li, Fengli Wang, Fei Wu, Jinrong Wang, Ling Liu, Changhua Lai

**Affiliations:** State Key Laboratory of Animal Nutrition, College of Animal Science and Technology, China Agricultural University, Beijing, 100193 China; College of Bio-engineering, Henan University of Technology, Zhengzhou, 450001, Henan China

**Keywords:** Digestible energy, Double-low rapeseed meal, Growing pigs, Ileal digestibility of amino acids, Metabolizable energy

## Abstract

**Background:**

The nutritional value of rapeseed meal may be variable due to the variation of its chemical composition. And a precise understanding of the nutritional value of an ingredient is beneficial for the accurate diet formulation and reduction of feed costs. This study was conducted to determine the chemical composition, digestible energy (DE) and metabolizable energy (ME) content, and apparent ileal digestibility (AID) and standardized ileal digestibility (SID) of amino acids (AA) for growing pigs. Thirteen solvent-extracted double-low rapeseed meal (DLRSM) samples were obtained from the main double-low rapeseed producing areas in China.

**Methods:**

The DE and ME contents of the 13 DLRSM samples were measured in growing pigs (six pigs per DLRSM sample, average initial body weight (BW) = 48.3 kg). The AID and SID of AA of 10 DLRSM samples were determined in 12 crossbred barrows (average initial BW = 35.3 kg) by using two 6 × 6 Latin square designs. Each Latin square comprised one N-free diet and 5 DLRSM test diets.

**Results:**

The chemical composition of DLRSM varied among samples, and the coefficient of variation was greater than 10 % for ether extract (EE), neutral detergent fiber (NDF), acid detergent fiber (ADF), calcium (Ca), and total glucosinolates. The AA content of DLRSM varied among samples especially for lysine (Lys) and methionine (Met). On a dry matter (DM) basis, the apparent total tract digestibility (ATTD) of gross energy (GE), the DE and ME and the ME:DE ratio of DLRSM averaged 62.39 %, 2862 kcal/kg and 2723 kcal/kg, and 94.95 %, respectively. The mean value of SID of Lys was 70.52 % which varied from 66.54–76.54 %. The SID of crude protein (CP), Met, and threonine (Thr) averaged 72.81 %, 82.41 %, and 69.76 %, respectively.

**Conclusions:**

There was great variability in chemical composition especially in the concentration of EE, NDF and ADF, but no significant differences in energy content of the DLRSM samples were observed. In addition, the AID and SID of all AA were relatively similar among DLRSM samples except for that of Lys.

## Background

Double-low rapeseed meal (DLRSM), a by-product of solvent-extracted rapeseed oil production, is a potential source of vegetable protein for use in swine diets. Double-low rapeseed contains less than 2 % erucic acid in the oil and less than 30 μmol/g glucosinolates in the meal, whereas rapeseed contains 25–45 % erucic acid in the oil and 50–100 μmol/g glucosinolates in the meal [1]. The superiority of DLRSM over rapeseed meal as a protein supplement is well known [2].

The nutritional value of DLRSM has been discussed [2], determined in many countries [3–6] and also included in the NRC [7]. However, the nutritional value of DLRSM in China is expected to be different compared with that of other countries due to the variation in varieties, seed quality, soil conditions, and processing conditions [1].

Although the variety of DLRSM in China is almost always *Brassica napus*, the difference within the species may lead to the variation of the quality of DLRSM. On the other hand, the temperature and time control in processing especially during desolventizing and toasting vary among different DLRSM plants in China; the heat treatment during this stage is critical for the quality of protein in DLRSM [8, 9]. In China, several researches for the nutritional value of rapeseed meal have been done [10–12], whereas little research has been done on DLRSM. So more information on the nutritional value of DLRSM produced in China is needed for diet formulation. Therefore, the objective of this study was to investigate the variation of the chemical composition, energy content and ileal amino acids (AA) digestibility of DLRSM.

## Methods

### General

All animal experimentation procedures were approved by the Institutional Animal Care and Use Committee at China Agricultural University (Beijing, China). Two experiments were conducted in the Metabolism Laboratory of the Ministry of Agriculture Feed Industry Centre (China Agricultural University, Beijing, China). The DLRSM samples were obtained from the main double-low rapeseed producing areas in China. All samples were solvent-extracted DLRSM. Thirteen meals were selected from the collected 20 DLRSM to achieve the greatest variability (Table 1). However, only 10 of the collected 20 DLRSM were selected to determine their ileal AA digestibility. The chemical composition and AA content of these DLRSM are shown in Tables 2 and 3. The diets in both experiments were all fed as mash. The pigs were fed the diets equal to 4 % of their body weight (BW) and the daily feed allowance [13] was offered in two equal meals at 0800 and 1700 h. Water was freely available at all times throughout the experiment. The barrows were individually placed in stainless-steel metabolism crates (1.4 m × 0.45 m × 0.6 m) in a temperature-controlled room (22 ± 2 °C). The pigs used in both experiments were crossbred (Duroc × Landrace × Yorkshire) barrows obtained from Fengning Swine Research Unit, China Agricultural University (Beijing, China).

**Table 1:** Source of double-low rapeseed meals used in the two experiments

Number	City and Province in China
1	Quyuan, Hunan
2	Lixian, Hunan
3	Luliang, Hunan
4	Xidongting, Hunan
5	Yingcheng, Hunan
6	Yiyang, Hunan
7	Huaihua, Hunan
8	Xiaodukou, Hunan
9	Feixi, Anhui
10	Jingzhou, Hubei
11	Yichang, Hubei
12	Gong’an, Hubei
13	Huanggang, Hubei

**Table 2:** Analyzed proximate composition (% of dry matter, unless otherwise indicated) of double-low rapeseed meals

Item	Double-low rapeseed meal number^a^																
	1	2	3	4	5	6	7	8	9	10	11	12	13	Mean	Maximum	Minimum	CV^b^
Dry matter	90.14	90.06	89.90	88.71	89.16	89.90	90.11	90.30	89.89	89.82	89.28	89.19	90.66	89.78	90.66	88.71	0.61
Crude protein	42.67	43.59	41.35	43.64	42.26	42.55	42.97	43.29	39.57	43.02	40.29	41.48	39.37	42.00	43.64	39.37	3.51
Ether extract	1.52	0.70	1.32	1.59	1.47	1.62	1.67	1.27	1.98	1.37	1.79	1.42	1.73	1.50	1.98	0.70	20.88
Crude fiber	15.15	15.14	14.48	12.73	13.50	15.35	17.26	13.16	12.60	13.41	14.81	14.15	15.08	14.37	17.26	12.6	9.03
Neutral detergent fibre	40.35	40.33	35.31	29.90	32.20	27.91	39.46	39.24	38.74	39.21	40.98	31.31	38.13	36.39	40.98	27.91	12.41
Acid detergent fibre	28.20	27.64	21.29	22.30	22.25	18.80	24.24	27.36	23.92	21.08	25.84	23.85	22.61	23.80	28.20	18.8	11.88
Ash	8.78	8.28	8.53	7.72	8.05	7.15	7.92	9.13	9.63	8.48	8.18	8.09	10.06	8.46	10.06	7.15	9.31
Calcium	0.94	0.90	0.98	0.83	0.88	0.81	0.74	0.92	0.69	1.08	0.79	0.95	0.90	0.88	1.08	0.69	12.05
Phosphorus	0.90	0.86	0.93	0.95	0.89	0.97	0.92	0.93	0.82	0.95	0.86	1.04	0.89	0.92	1.04	0.82	6.33
Gross energy, kcal/kg	4575	4568	4542	4636	4585	4707	4650	4557	4499	4587	4592	4645	4508	4589	4707	4499	1.28
Total glucosinolates, μmol/g	5.57	6.58	5.11	4.15	9.83	7.02	16.83	10.04	5.29	20.08	6.59	22.73	11.11	10.07	22.73	4.15	60.37

^a^Sources of double-low rapeseed meal are described in Table 1

^b^ *CV* coefficient of variation

**Table 3:** Analyzed AA composition of double-low rapeseed meals (% of dry matter)

Item	Double-low rapeseed meal number^a^												
	1	2	3	4	5	8	10	11	12	Mean	Maximum	Minimum	CV^b^
Crude protein	42.67	43.59	41.35	43.64	42.26	43.29	43.02	40.29	41.48	42.40	43.64	40.29	2.72
Indispensable AA													
Arg	2.18	2.23	2.14	2.41	2.31	2.19	2.27	2.08	2.22	2.23	2.41	2.08	4.35
His	1.05	1.06	1.04	1.20	1.14	1.13	1.13	1.06	1.09	1.10	1.20	1.04	4.83
Ile	1.52	1.51	1.42	1.56	1.51	1.51	1.48	1.38	1.41	1.48	1.56	1.38	4.03
Leu	2.67	2.67	2.55	2.76	2.65	2.65	2.68	2.47	2.54	2.63	2.76	2.47	3.38
Lys	1.96	1.94	2.02	2.41	2.25	1.97	2.06	1.95	2.21	2.09	2.41	1.94	7.92
Met	0.86	0.91	0.94	0.97	0.91	0.94	1.00	0.77	0.76	0.90	1.00	0.76	9.46
Phe	1.34	1.36	1.28	1.36	1.31	1.31	1.31	1.22	1.27	1.31	1.36	1.22	3.59
Thr	1.84	1.86	1.80	1.91	1.79	1.79	1.86	1.76	1.81	1.82	1.91	1.76	2.50
Trp	0.51	0.54	0.50	0.53	0.52	0.51	0.52	0.46	0.50	0.51	0.54	0.46	4.49
Val	2.34	2.35	2.17	2.38	2.28	2.28	2.24	2.15	2.19	2.26	2.38	2.15	3.66
Dispensable AA													
Ala	1.75	1.75	1.76	2.05	1.96	1.97	1.98	1.86	1.90	1.89	2.05	1.75	5.95
Asp	2.77	2.82	2.74	2.93	2.80	2.82	2.87	2.60	2.72	2.79	2.93	2.60	3.42
Cys	1.15	1.18	1.19	1.24	1.22	1.11	1.19	1.14	1.15	1.17	1.24	1.11	3.60
Glu	6.98	7.01	6.68	7.41	7.12	7.16	7.30	6.67	6.82	7.02	7.41	6.67	3.73
Gly	2.51	2.52	2.30	2.36	2.26	2.27	2.32	2.15	2.21	2.32	2.52	2.15	5.38
Pro	2.25	2.10	2.05	2.46	2.35	2.33	2.36	2.24	2.30	2.27	2.46	2.05	5.76
Ser	1.71	1.73	1.66	1.75	1.65	1.63	1.73	1.62	1.66	1.68	1.75	1.62	2.81
Tyr	0.74	0.77	0.68	0.81	0.77	0.76	0.76	0.76	0.75	0.75	0.81	0.68	5.36

^a^Sources of double-low rapeseed meal are described in Table 1

^b^ *CV* coefficient of variation

### Exp. 1: Energy digestibility

This experiment was conducted to determine the apparent total tract digestibility (ATTD) of gross energy (GE), the digestible energy (DE) and metabolizable energy (ME) and the ME:DE ratio of 13 DLRSM fed to growing pigs. Eighty-four crossbred barrows (initial BW: 48.3 ± 2.4 kg; Duroc × Landrace × Yorkshire) were allocated to one of 14 dietary treatments in a completely randomized design with 6 barrows in each dietary treatment. The 14 experimental diets included one corn-soybean meal basal diet and 13 DLRSM test diets. The DLRSM test diets were formulated to contain 19.2 % DLRSM which replaced 20 % of the energy supplied by corn, soybean meal, and Lys in the basal diet (Table 4). The chemical composition of the experimental diets is presented in Table 5.

**Table 4:** Ingredient composition of experimental diets (as-fed basis, %)

Item	Exp. 1		Exp. 2	
	Basal diet	Test diets	Double-low rapeseed meal diets	N-free diet
Corn	77.40	61.90	-	-
Soybean meal	18.60	14.90	-	-
Double-low rapeseed meal	-	19.20	40.00	-
Cornstarch	-	-	34.40	68.90
Sucrose	-	-	20.00	20.00
Cellulose acetate^a^	-	-	-	4.00
Soybean oil	-	-	3.00	3.00
L-lysine · HCl^b^	0.10	0.08	-	-
Dicalcium phosphate	1.20	1.20	1.00	1.60
Potassium carbonate	-	-	-	0.30
Magnesium oxide	-	-	-	0.10
Limestone	1.10	1.10	0.50	1.00
Wheat rice stone^c^	0.80	0.80	-	-
Vitamin-mineral premix^d^	0.50	0.50	0.50	0.50
Salt	0.30	0.30	0.30	0.30
Chromic oxide	-	-	0.30	0.30

^a^Made by Chemical Reagents Company, Beijing, China

^b^Made by Dacheng Group, Jilin, China

^c^Used as carrier for L-lysine · HCl and contained more than 70 % silicon oxide and aluminium oxide, made by YiXian BeiQiao Tou Ore Company (YiXian, China)

^d^Provided the following quantities of vitamins and minerals per kg of complete diet: Mn, 50 mg (MnO); Fe, 125 mg (FeSO_4_ · H_2_O); Zn, 125 mg (ZnO); Cu, 150 mg (CuSO_4_ · 5H_2_O); I, 50 mg (CaI_2_); Se, 0.30 mg (Na_2_SeO_3_), retinyl acetate, 4500 IU; cholecalciferol, 1350 IU; DL-α-tocopheryl acetate, 13.5 mg; menadione sodium bisulfite complex, 2.7 mg; niacin, 18 mg; vitamin B_12_, 27.6 μg; thiamine, 0.6 mg; pyridoxine, 0.9 mg; riboflavin, 1.8 mg; D-calcium-pantothenate, 10.8 mg; nicotinic acid, 30.3 mg; choline chloride, 210 mg

**Table 5:** Analyzed composition of the experimental diets used in Exp. 1 (% as-fed basis)

Diet	Basal diet	Test diets^a^												
		1	2	3	4	5	6	7	8	9	10	11	12	13
Dry matter	89.15	89.66	89.64	89.64	89.41	89.65	89.74	89.87	89.79	90.05	89.53	89.50	89.46	89.60
Crude protein	15.19	19.13	19.06	18.78	19.59	19.29	18.91	19.52	19.61	18.90	19.40	18.51	18.70	18.77
Ether extract	1.98	2.34	2.20	2.12	1.84	1.86	1.93	2.02	1.94	1.88	2.10	2.05	2.01	1.94
Crude fiber	1.95	4.30	4.41	4.31	4.00	4.11	3.94	4.01	4.05	4.27	4.07	4.26	4.29	4.01
Neutral detergent fibre	10.14	18.99	15.31	15.41	14.90	15.51	15.21	16.75	16.28	16.76	16.17	16.31	15.35	16.48
Acid detergent fibre	3.12	8.36	6.11	6.90	6.73	6.87	6.57	6.85	7.13	7.39	6.92	7.25	7.07	7.07
Ash	5.46	6.51	6.28	6.33	6.09	6.34	6.26	6.13	6.49	6.48	6.41	6.30	6.31	6.97
Calcium	0.64	0.79	0.77	0.77	0.76	0.73	0.69	0.67	0.78	0.69	0.81	0.36	0.82	0.79
Phosphorus	0.58	0.65	0.68	0.69	0.68	0.69	0.68	0.67	0.68	0.68	0.69	0.33	0.69	0.69
Gross energy, kcal/kg	3786	3817	3841	3826	3830	3816	3836	3829	3820	3815	3818	3823	3821	3797

^a^Sources of double-low rapeseed meal are described in Table 1

The experiment lasted 14 d which comprised 9-d for adaptation to the diets followed by a 5-d total collection of feces and urine. Feces were collected into bags (one pig per bag) immediately when it appeared in the metabolism crates and stored at −20 °C. Urine was collected in buckets located under the metabolism crates. The buckets contain 50 mL of 6 *N* HCl and the urine was measured by volume every morning. A sample (10 % of the total volume) was collected, and after filtering, the urine samples were stored at −20 °C. The collection procedures were conducted according to the methods described by Song et al. [14] and Ren et al. [15].

### Exp. 2: Amino acid digestibility

This experiment was conducted to determine the apparent ileal digestibility (AID) and standardized ileal digestibility (SID) of crude protein (CP) and AA in 10 samples of DLRSM (numbers 1–5, 8, 10–12; Table 1) fed to growing pigs. Twelve crossbred barrows (initial BW: 35.3 ± 3.8 kg; Duroc × Landrace × Yorkshire) with a T-cannula near the distal ileum were assigned to one of two 6 × 6 Latin square designs. Each Latin square comprised one N-free diet and 5 DLRSM test diets. The test diets contained 40 % of one of the 10 DLRSM as the sole source of protein, and the N-free diet was formulated according to the methods described by Stein et al. [16] to estimate the basal endogenous losses of CP and AA for determining the SID of CP and AA (Table 4). The chemical composition of the Exp. 2 diets is presented in Table 6. The procedure of equipping the pig with a T-cannula near the distal ileum was according to the method described by Stein et al. [17]. (1998). Each of the six periods in the Latin square design lasted 7 d within which the first 5 d were for adaptation and the last 2 d for ileal digesta collection. Ileal digesta were collected continuously from 0800–1700 h. The collection procedures used followed the description provided by Stein et al. [17]. Briefly, on d 6 and 7, a 200 mL plastic bag was attached to the open cannula using a cable tie. The bags were removed whenever they were filled with digesta or at least every 30 min and stored at −20 °C until required for analysis.

**Table 6:** Analyzed composition of the experimental diets used in Exp. 2 (% as-fed basis)

Item	Double-low rapeseed meal diets^a^									N-free diet
	1	2	3	4	5	8	10	11	12	
Dry matter	93.50	92.65	92.78	92.70	93.21	93.17	93.17	93.16	92.89	92.45
Crude protein	16.40	16.11	15.34	15.33	15.92	15.73	15.23	14.64	15.29	0.87
Indispensable AA										
Arg	0.84	0.97	0.82	0.85	0.75	0.75	0.76	0.62	0.73	-
His	0.40	0.46	0.39	0.41	0.41	0.40	0.39	0.38	0.38	-
Ile	0.69	0.75	0.66	0.64	0.64	0.65	0.63	0.59	0.63	-
Leu	1.07	1.18	1.02	1.03	0.98	1.00	1.00	0.90	0.96	-
Lys	0.72	0.83	0.75	0.83	0.82	0.71	0.75	0.71	0.80	-
Met	0.26	0.27	0.25	0.25	0.27	0.24	0.26	0.23	0.25	-
Phe	0.56	0.65	0.53	0.54	0.60	0.56	0.55	0.57	0.56	-
Thr	0.63	0.73	0.63	0.65	0.65	0.64	0.62	0.61	0.63	-
Trp	0.18	0.18	0.16	0.17	0.19	0.18	0.18	0.18	0.17	-
Val	0.80	0.89	0.74	0.74	0.83	0.77	0.79	0.77	0.78	-
Dispensable AA										
Ala	0.66	0.75	0.62	0.64	0.69	0.66	0.67	0.62	0.66	-
Asp	1.01	1.16	1.00	1.02	1.03	1.02	1.00	0.90	0.98	-
Cys	0.36	0.36	0.35	0.32	0.37	0.30	0.33	0.30	0.33	-
Glu	2.55	2.93	2.44	2.50	2.55	2.51	2.43	2.30	2.41	-
Gly	0.74	0.84	0.71	0.72	0.75	0.73	0.72	0.68	0.71	-
Pro	0.93	1.07	0.99	0.87	0.88	0.93	0.86	0.82	0.87	-
Ser	0.59	0.70	0.60	0.63	0.61	0.62	0.61	0.56	0.60	-
Tyr	0.76	0.81	0.77	0.75	0.69	0.68	0.71	0.67	0.68	-

^a^Sources of double-low rapeseed meal are described in Table 1

### Sample preparation and chemical analyses

Fecal samples were dried in an oven at 65 °C for three days, weighed, pooled for each pig and subsampled. Ileal digesta samples were thawed, mixed within pig and period, subsampled and lyophilized in a vacuum-freeze dryer (Tofflon Freezing Drying Systems, Shanghai, China). Samples of DLRSM, diets, feces, and ileal digesta were ground through a 1-mm screen and thoroughly mixed for analysis. Urine samples were thawed and thoroughly mixed within pig for analysis.

Samples of DLRSM, diets, feces, and digesta was analysed for dry matter (DM) (procedure 930.15) [18]. All DLRSM samples and diets in Exp. 1 were analyzed for CP (procedure 984.13), ash (procedure, 942.05), calcium (procedure 968.08), phosphorus (procedure 946.06), and crude fiber (CF) (procedure 978.10) [18]. Ether extract (EE) were determined according to the method of Thiex et al. [19] and Kjeldahl N was determined according to the method of Thiex et al. [20]. The concentrations of neutral detergent fiber (NDF) and acid detergent fiber (ADF) were determined according to the method of Van Soest et al. [21]. The concentration of NDF was analyzed using heat-stable α-amylase and sodium sulfite without correction for insoluble ash as adapted for an Ankom Fiber Analyzer (Ankom Technology, Macedon, NY). The ADF fraction was analyzed in a separate sample. The concentration of total glucosinolates was analyzed according to Daun et al. [22]. Samples of DLRSM, diets, feces, and urine were analyzed for gross energy via an adiabatic oxygen bomb calorimeter (Parr Instruments Co., Moline, IL).

Samples of DLRSM, diets, and ileal digesta from Exp. 2 were hydrolyzed with 6 *N* HCl for 24 h at 110 °C [18] and analyzed for 15 AA using an Amino Acid Analyzer (Hitachi L-8900, Tokyo, Japan). The sulfur-containing AA, methionine (Met) and cysteine (Cys), were subjected to performic acid oxidation and hydrolyzed with 7.5 *N* HCl for 24 h at 110 °C [18] before measurement using an Amino Acid Analyzer (Hitachi L-8800, Tokyo, Japan). Tryptophan (Trp) was determined after LiOH hydrolysis for 22 h at 110 °C [18] using High Performance Liquid Chromatography (Agilent 1200 Series, Santa Clara, CA). The chromium (Cr) concentration of diets and ileal digesta samples was determined after nitric acid-perchloric acid wet ash sample preparation using a Polarized Zeeman Atomic Absorption Spectrometer (Hitachi Z2000, Tokyo, Japan).

### Calculations

In Exp. 1, the DE and ME of 13 DLRSM were determined. The ATTD of GE as well as DE and ME content of the DLRSM were calculated using the difference method [13].

In Exp. 2, the AA digestibility of DLRSM samples was calculated as described by Stein et al. [16]. Because DLRSM was the only feed ingredient contributing AA in the experimental diets, dietary values also represent the digestibility for each sample of DLRSM. The AID of AA in the diets containing DLRSM was calculated according to the following equation:

$$ \mathrm{AID} = \left[1 - \left(A{A}_{\mathrm{digesta}}/A{A}_{\mathrm{diet}}\right) \times \left(C{r}_{\mathrm{diet}}/C{r}_{\mathrm{digesta}}\right)\right] \times 100\%, $$

where AID is the apparent ileal digestibility of an AA (%), AA_digesta_ is the AA concentration in the ileal digesta (g/kg of DM), AA_diet_ is the AA concentration in the diets (g/kg of DM), Cr_diet_ is the chromium concentration in the diet (g/kg of DM), and Cr_digesta_ is the chromium concentration in the ileal digesta (g/kg of DM). The AID of CP was calculated using the equation above.

The IAA_end_ of AA for each pig fed the N-free diet was determined according to following equation:

$$ IA{A}_{\mathrm{end}} = \left[A{A}_{\mathrm{digesta}} \times \left(C{r}_{\mathrm{diet}}/C{r}_{\mathrm{digesta}}\right)\right], $$

where IAA_end_ is the basal ileal endogenous loss of an AA (g/kg of DM intake). The endogenous loss of CP was also determined using the same equation.

By correcting the AID of each AA, which was calculated for each sample for the IAA_end_ of each AA, the SID of AA was corrected according to following equation:

$$ SID = \left[\mathrm{AID} + \left(IA{A}_{\mathrm{end}}/A{A}_{\mathrm{diet}}\right) \times 100\right], $$

where SID is the standardized ileal digestibility of an AA (%).

### Statistical analyses

The data for the ATTD of GE, the DE and ME content, the ME:DE ratio were analyzed using the GLM procedure (SAS Institute Inc., Cary, NC), with the pig as the experimental unit. The data on AID and SID were analyzed using the ANOVA procedure with DLRSM source, pig, and period as the main effects. The total variation was analyzed by the model described by Ji et al. [23]. In all analyses, the differences were considered significant if *P* < 0.05.

## Results

### Chemical composition of DLRSM

The chemical composition of the 13 DLRSM is presented in Table 2, and the chemical composition of the experimental diets is presented in Table 5. The chemical composition of DLRSM varied among samples, whereas the coefficient of variation was greater than 10 % for ether extract, NDF, ADF, Ca, and total glucosinolates. The content of CF and ash were also quite variable. The DM content of 13 DLRSM averaged 89.78 %, ranged from 88.71–90.66 %. On a DM basis, the total glucosinolates content (μmol/g) ranged from 4.15–22.73 with an average of 10.07.

### Exp. 1: Energy digestibility

The ATTD of GE, the DE and ME and ME:DE ratio of the 13 DLRSM are presented in Table 7. There were no differences in these values among the different DLRSM. On a DM basis, the DE content of the DLRSM varied by 483 kcal/kg and ranged from 2616–3099 kcal/kg; the ME content of the DLRSM varied by 450 kcal/kg and ranged from 2514–2964 kcal/kg. The ATTD of GE and the ME:DE ratio averaged 62.39 and 94.95 %, with a range of 57.41–68.75 %, and 91.70–97.41 %, respectively.

**Table 7:** Energy concentration and apparent total tract digestibility (ATTD) of GE of double-low rapeseed meals fed to growing pigs (Exp. 1, DM basis)

Item	ATTD of GE, %	DE, kcal/kg	ME, kcal/kg	ME/DE, %
Number^a^				
1	65.81	3011	2921	97.41
2	63.20	2887	2720	94.16
3	64.65	2936	2814	95.88
4	58.86	2729	2534	92.58
5	58.35	2675	2522	93.84
6	61.44	2892	2769	95.50
7	61.99	2882	2689	92.97
8	57.41	2616	2537	97.05
9	58.24	2620	2514	95.59
10	64.16	2943	2819	95.23
11	61.58	2828	2733	96.64
12	66.58	3093	2964	95.83
13	68.75	3099	2868	91.70
SEM	3.32	151.51	173.75	2.42
Mean	62.39	2862	2723	94.95
Maximum	68.75	3099	2964	97.41
Minimum	57.41	2616	2514	91.70
CV^b^	5.69	5.69	5.74	1.87
*P*-value	0.31	0.30	0.63	0.88

^a^Sources of double-low rapeseed meal are described in Table 1

^b^ *CV* coefficient of variation

### Exp. 2: Amino acid digestibility

The data on one of the 10 DLRSM (DLRSM number 13) was considered an outlier because the value was more than three standard deviations away from the mean [24]. Therefore, only 9 of the 10 DLRSM are discussed here. The chemical composition of the DLRSM (numbers 1–5, 8, 10–12) is shown in Table 2, and their AA content (%) is shown in Table 3. As expected, the AA content of DLRSM varied among samples especially for Lys and Met. The level (%) of CP ranged from 40.29–43.64 with an average of 42.40 on a DM basis. The concentrations of Lys and Met of DLRSM ranged from 1.94–2.41 % and 0.76–1.00 % with an average of 2.09 % and 0.90 %, respectively.

Tables 8 and 9 show the AID and SID values of CP and AA, respectively. There were no significant differences in the AID of CP and AA (except for Lys) among different DLRSM, but the largest variation among indispensable AA was Lys, and among dispensable AA was Pro. Also there were no significant differences in the SID of CP and AA (except for Lys) among different DLRSM, the largest variation among indispensable AA was for Lys, and among the dispensable AA, the greatest variation was observed for Pro. The AID values of Lys ranged from 60.41–68.53 % with an average of 63.85 %, and SID values ranged from 68.69–76.72 % with an average value of 72.81 %.

**Table 8:** Apparent ileal digestibility (%) of AA in double-low rapeseed meals fed to growing pigs (Exp. 2)

Item	Double-low rapeseed meal number^a^														
	1	2	3	4	5	8	10	11	12	SEM	Mean	Maximum	Minimum	CV^b^	*P*-value
Crude protein	61.57	60.41	62.44	67.32	66.67	60.71	63.35	63.65	68.53	1.98	63.85	68.53	60.41	4.67	0.18
Indispensable AA															
Arg	79.95	82.57	79.73	82.58	80.44	77.16	78.15	77.55	80.80	1.82	79.88	82.58	77.16	2.49	0.61
His	78.61	78.95	79.69	81.92	82.22	75.51	81.02	80.62	81.00	1.36	79.95	82.22	75.51	2.59	0.17
Ile	73.13	76.16	74.67	74.72	74.90	71.56	76.18	73.89	75.80	2.31	74.56	76.18	71.56	2.03	0.97
Leu	73.57	76.96	75.86	76.01	75.17	71.27	74.32	73.59	75.66	2.00	74.71	76.96	71.27	2.31	0.85
Lys	62.48	65.10	65.61	71.63	69.04	62.63	67.91	63.25	73.08	1.90	66.75	73.08	62.48	5.85	0.01
Met	77.76	80.26	79.43	79.71	83.22	76.41	80.90	79.49	81.58	1.50	79.86	83.22	76.41	2.51	0.32
Phe	72.08	77.70	73.44	74.21	77.27	73.53	74.64	76.68	76.35	1.69	75.10	77.70	72.08	2.62	0.52
Thr	57.68	64.47	62.72	65.75	64.77	60.33	62.70	62.67	66.81	2.50	63.10	66.81	57.68	4.45	0.57
Trp	69.44	67.08	69.33	71.56	72.94	66.64	70.53	71.53	73.64	3.50	70.30	73.64	66.64	3.44	0.74
Val	67.45	71.68	68.48	69.61	72.62	66.48	70.50	71.66	72.91	2.74	70.15	72.91	66.48	3.28	0.48
Dispensable AA															
Ala	65.55	69.75	66.14	71.26	72.15	65.28	69.26	69.40	73.48	2.04	69.14	73.48	65.28	4.27	0.23
Asp	61.78	63.91	66.89	66.73	66.14	60.42	63.90	63.28	67.62	3.55	64.52	67.62	60.42	3.85	0.62
Cys	66.78	60.72	65.15	65.76	69.31	57.82	63.98	60.76	70.15	4.55	64.49	70.15	57.82	6.38	0.26
Glu	76.60	80.66	79.38	81.47	81.70	76.66	79.12	79.95	81.86	1.73	79.71	81.86	76.60	2.51	0.22
Gly	55.26	57.12	54.06	64.67	64.26	53.30	58.27	58.20	64.78	5.12	58.88	64.78	53.30	7.80	0.17
Pro	48.74	51.95	44.28	61.23	56.24	45.30	40.05	50.26	63.92	10.32	51.33	63.92	40.05	15.44	0.19
Ser	59.98	66.46	64.59	68.15	66.61	63.13	65.63	63.72	69.24	3.64	65.28	69.24	59.98	4.29	0.50
Tyr	82.21	82.68	82.41	81.32	80.47	77.93	82.53	80.38	82.00	4.55	81.33	82.68	77.93	1.89	0.99

^a^Sources of double-low rapeseed meal are described in Table 1

^b^ *CV* coefficient of variation

**Table 9:** Standardized ileal digestibility (%) of AA in double-low rapeseed meals fed to growing pigs (Exp. 2)^a,b^

Item	Double-low rapeseed meal number^a^														
	1	2	3	4	5	8	10	11	12	SEM	Mean	Maximum	Minimum	CV^c^	*P*-value
Crude protein	71.19	70.12	72.65	75.46	74.55	68.69	73.68	72.22	76.72	1.94	72.81	76.72	68.69	3.55	0.33
Indispensable AA															
Arg	87.56	89.10	87.41	87.76	86.36	83.03	86.44	84.69	86.88	1.94	86.58	89.10	83.03	2.07	0.80
His	82.74	82.56	83.99	85.07	85.34	78.69	85.32	83.98	84.36	1.51	83.56	85.34	78.69	2.50	0.28
Ile	77.03	79.71	78.71	77.61	77.80	74.42	80.39	77.05	78.72	2.38	77.94	80.39	74.42	2.24	0.94
Leu	78.38	81.28	80.83	80.08	79.47	75.48	79.44	78.27	80.04	1.64	79.25	81.28	75.48	2.18	0.65
Lys	66.71	68.72	69.64	74.97	72.45	66.54	71.92	67.16	76.54	1.70	70.52	76.54	66.54	5.20	0.01
Met	80.26	82.67	81.98	82.27	85.64	79.10	83.42	82.25	84.14	1.39	82.41	85.64	79.10	2.36	0.26
Phe	76.44	81.43	78.01	78.54	81.18	77.72	79.06	80.83	80.56	1.62	79.31	81.43	76.44	2.23	0.61
Thr	65.27	70.93	70.22	71.76	70.79	66.43	70.36	69.08	73.02	2.29	69.76	73.02	65.27	3.56	0.62
Trp	75.14	72.56	75.46	76.97	77.90	71.91	76.10	76.81	78.98	2.32	75.76	78.98	71.91	3.07	0.71
Val	72.27	75.98	73.62	73.84	76.43	70.58	75.39	75.71	76.93	1.73	74.53	76.93	70.58	2.83	0.45
Dispensable AA															
Ala	74.38	77.49	75.49	78.47	78.94	72.32	77.98	76.95	80.51	2.01	76.95	80.51	72.32	3.26	0.42
Asp	66.57	70.50	70.50	71.97	71.34	65.71	70.07	69.22	73.08	2.17	69.88	73.08	65.71	3.44	0.59
Cys	72.21	66.20	70.74	73.12	75.74	65.60	69.94	68.56	77.39	2.37	71.06	77.39	65.60	5.64	0.08
Glu	79.50	83.16	82.39	84.03	84.22	79.22	82.14	82.75	84.53	1.31	82.44	84.53	79.22	2.34	0.21
Gly	78.19	77.18	77.89	82.40	81.42	70.83	81.90	77.03	82.74	3.94	78.84	82.74	70.83	4.82	0.76
Pro	102.94	98.73	94.78	101.78	96.57	83.59	98.57	93.55	104.84	14.57	97.26	104.84	83.59	6.52	0.99
Ser	67.86	73.09	72.30	74.23	72.92	69.33	73.32	70.57	75.61	2.18	72.14	75.61	67.86	3.40	0.57
Tyr	89.31	89.29	89.42	85.58	85.14	82.67	90.12	85.17	86.76	2.39	87.05	90.12	82.67	2.98	0.62

^a^Sources of double-low rapeseed meal are described in Table 1

^b^Values for standardized ileal digestibility were calculated by correcting apparent ileal digestibility for the basal endogenous losses. Basal ieal endogenous were determined (g/kg dry matter intake) as crude protein, 15.2; Arg, 0.58; His, 0.16; Ile, 0.24; Leu, 0.50; Lys, 0.31; Met, 0.07; Phe, 0.26; Thr, 0.47; Trp, 0.10; Val, 0.37; Ala, 0.56; Asp, 0.62; Cys, 0.23; Glu, 0.74; Gly, 1.60; Pro, 4.61; Ser, 0.46; Tyr 0.46

^c^ *CV* coefficient of variation

## Discussion

The objectives of this study were to investigate the variation of the chemical composition, energy content and ileal AA digestibility of DLRSM. The pigs used in the two experiments remained healthy and had no feed refusals throughout the experiment.

The chemical composition and AA content of DLRSM varied among samples, which may be due to different double-low rapeseed sources and processing techniques [1]. All DLRSM samples used in this experiment were solvent-extracted *Brassica napus*, but there may be considerable variation in the temperatures used during double-low rapeseed processing. From Table 2, it appears that the total glucosinolates content varied among samples, which may be a result of differences among varieties of rapeseeds. It is also possible that other cruciferous crops caused cross pollination in the double low rapeseeds and thereby reduced the quality. Variation in the oil content among DLRSM (0.70–1.98 %) may result from the differences in processing conditions during the oil extraction among different crushing plants. The NDF content of 13 kinds of DLRSM was large and variable, this may be because of different degree of Maillard reactions during desolventization and toasting as reported by Woyengo et al. [5].

The concentration of CP in the 13 DLRSM sources was slightly greater than the values published by Fan et al. [25] and Landero et al. [26], but similar to the values reported by Bell and Keith [27], Xi et al. [11] and Woyengo et al. [5]. The AA composition (except Met and Cys) of the 13 DLRSM sources was slightly lower than the values published by Fan et al. [25], Woyengo et al. [5], and Landero et al. [26], and the NDF content of the 13 DLRSM sources was greater than the values published by these studies, but similar to the values reported by Xi et al. [11], using DLRSM also produced in China. The reason why the NDF content in this study were greater than previously published values may be due to differences in variety, the different analytical methods used [28] or a result of Maillard reactions during desolventization and toasting as reported by Woyengo et al. [5]. In addition, Maillard reactions may have reduced concentrations of AA in DLRSM. That is the reason why the AA composition (except Met and Cys) of the 13 DLRSM sources in the current study were slightly lower and the NDF content was greater than previous data. The greater content of S-containing AA in our DLRSM may due to that the samples in the current study underwent the desolventization and toasting with longer time or higher temperature, because Newkirk et al. [29] reported the desolventization and toasting increased the content of S-containing AA. The content of ether extract in the 13 DLRSM sources was lower than the values reported by Fan et al. [25], NRC [7], Woyengo et al. [5], and Landero et al. [26]. The lower content of ether extract may be due to the improved oil extraction methods for the DLRSM used in this study or due to no gums being added to the meals, which caused a lower GE content.

Though DLRSM is a protein supplement, its available energy content is critical for feed formulation [1]. Although the difference of energy content among DLRSM samples was not significant, the gap between the highest value and the lowest value was so large that it couldn’t be ignored during the feed formulation. The difference in energy content among the DLRSM samples may be caused by the different total glucosinolates content, fiber content, and protein content. The reduction of total glucosinolates improves the available energy content of DLRSM [30], and reduced fiber concentration and greater protein concentration increases the concentration of gross energy [1]. The average DE for the 13 DLRSM (2862 kcal/kg on a DM basis) was lower than the values (3584 kcal/kg on a DM basis) reported by NRC [7], but was similar to the DE published by Whittemore et al. [31], and within the range of DE values (2581–3346 kcal/kg on a DM basis) reported by de Lange et al. [32] The mean ME content for DLRSM in the present study (2723 kcal/kg on a DM basis) was lower than the value (3299 kcal/kg on a DM basis) published by NRC [7] but within the range of ME values (2701–3497 kcal/kg on a DM basis) reported by Bell [1]. The lower DE and ME values may be due to lower content of ether extract or greater fiber content of DLRSM used in the current study [1]. The average ME:DE ratio (94.95 %) for the 13 DLRSM agrees with the value reported by Sibbald [33], who concluded that the ME content of DLRSM was 86–96 % of DE content.

The AID of the essential AA in the present experiment was slightly lower than the values reported in NRC [7] and the SID of AA in the present experiment was slightly lower than the values reported by previous research [34, 35]. The decreased AA digestibility for DLRSM used in current study may be due to Maillard reaction that occurred during the desolventization and toasting process or the high fiber concentration in the DLRSM [1, 5]. During processing of DLRSM, a temperature of 80–90 °C is necessary to denature myrosinase [1, 9]. However, the temperature used in the stage of cooking, prepressing, desolventizing and toasting in actual production is usually higher than 100 °C. This level of heat may lead to Maillard reactions, which will result in reduced AA concentration [1, 36]. This may be also a factor for the difference of AID and SID values of Lys among the DLRSM samples in the present study.

In conclusion, there was variability in chemical composition especially the concentration of EE, NDF and ADF, but no significant differences in energy content of the DLRSM samples. In addition, the AID and SID of Lys vary among the DLRSM samples, but for most other AA, the AID and SID are relatively similar among different samples. Future work should be conducted to identify the relationship between the chemical composition and the nutritional value and determine the effects of the individual steps of process procedure on the energy content and AA digestibility in the DLRSM.

## Abbreviations

AA: Amino acid
ADF: Acid detergent fiber
AID: Apparent ileal digestibility
ATTD: Apparent total tract digestibility
BW: Body weight
CP: crude protein
CF: Crude fiber
CV: Coefficient of variation
DE: Digestible energy
DLRSM: Double-low rapeseed meal
EE: Ether extract
GE: Gross energy
ME: Metabolizable energy
NDF: Neutral detergent fiber
SEM: Standard error of the mean
SID: Standard ileal digestibility
